# Draft Genome Sequence of *Flavobacterium* *psychrophilum* Strain SSADA-1411, Isolated from an Ayu (*Plecoglossus altivelis altivelis*) Migrating Downriver To Spawn in the Shimanto River, Kochi, Japan

**DOI:** 10.1128/genomeA.00735-17

**Published:** 2017-08-03

**Authors:** Masayuki Imajoh, Yuto Tsuji, Hazuki Yamashita, Masayuki Ohgi, Shinya Monno, Kouhei Ohnishi, Kikuo Horioka

**Affiliations:** a Fish Disease Laboratory, Department of Aquaculture, Kochi University, Nankoku, Kochi, Japan; b Shimanto City Office, Shimanto, Kochi, Japan; c Shimantogawa Chuo Fisheries Cooperative Association, Shimanto, Kochi, Japan; d Research Institute of Molecular Genetics, Kochi University, Nankoku, Kochi, Japan

## Abstract

Here, we report the draft genome sequence and annotation of *Flavobacterium psychrophilum* strain SSADA-1411. This strain was isolated from the skin ulcer of an ayu (*Plecoglossus altivelis altivelis*) migrating downriver to spawn in the lower Shimanto River, in western Kochi Prefecture on Shikoku Island in Japan.

## GENOME ANNOUNCEMENT

*Flavobacterium psychrophilum*, of the family *Flavobacteriaceae* (1), order *Flavobacteriales* (2), and class *Flavobacteriia* (3), is the etiological agent of bacterial cold-water disease (BCWD) (4). This bacterium is classified into two genotypes, A and B, based on PCR-restriction fragment length polymorphism assay of the peptidyl-prolyl *cis*-*trans* isomerase C gene (5). Type A may be unique to ayu *Plecoglossus altivelis altivelis*, because isolates from salmonids, cyprinids, and Japanese eel *Anguilla japonica* are all type B. Also, some isolates from ayu harbor a 2.8-, 3.4-, or 4.1-kbp plasmid (6). Four genotypes—type G-C, type A-T, type A-C, and type G-T—are classified based on genotyping by single nucleotide polymorphism analysis of the DNA gyrase subunit A gene (7). Type G-C shows strong pathogenicity to ayu, whereas the other three exhibit no or weak pathogenicity.

The Shimanto River, in western Kochi Prefecture on Shikoku Island in Japan, is the longest river in Shikoku, with a length of 196 km, and is known as Japan’s last remaining limpid river. It was once famous for its abundance of ayu, which is a popular angling fish and an important food staple in Japan. However, the catch has been low since the mid-1990s, because BCWD has depressed the ayu population (8). Mature ayu migrate down to the lower reaches of the river in autumn every year, lay their eggs on the river bed, and die. Forty migrating ayu were caught with a cast net during the 2014 spawning season, of which eight (20%) had skin ulcers. We isolated strain SSADA-1411 from one of these ulcers and identified the isolate as type A/G-C, harboring a 4.1-kbp plasmid (data not shown).

The genomic DNA of SSADA-1411 was extracted, purified, and sequenced according to our previous method (9). Genome assembly was performed using GS De Novo Assembler v2.9 (Roche). The assembled strain SSADA-1411 consisted of 164 contigs (>500 bp), totaling 2,707,110 bp, with a G+C content of 32.5%. The genome sequence was annotated using Microbial Genome Annotation Pipeline (MiGAP) v2.21 (10). A total of 3,001 protein-coding sequences (CDS) were predicted, and at least 37 tRNA genes and three rRNA operons were identified. The Rapid Annotations in Subsystems Technology (RAST) server v2.0 (11) was used for subsystem descriptions. According to this analysis there were 320 subsystems but no subsystem features for photosynthesis or motility and chemotaxis. The RAST results were identical to those of our previously reported isolate, KTEN-1510, which had similar genotypes to SSADA-1411 and was isolated from the gill of a migrating ayu in the Kagami River, also in Kochi (9). The availability of draft genome sequences of these two isolates may be helpful for understanding the mechanisms underlying their high host specificity and pathogenicity to ayu.

### Accession number(s).

The draft genome sequence of strain SSADA-1411 has been deposited in GenBank under accession number BDSH00000000. The version described in this paper is the first version, BDSH01000000.
